# Women’s participation in a disease management intervention for podoconiosis in northern Rwanda: understanding the context of women’s lives

**DOI:** 10.1186/s12939-026-02795-1

**Published:** 2026-02-21

**Authors:** Corinna Thellmann, Gemma Aellah, Valerienne Maltemps, Gail Davey, Dieudonne Uwizeye, Papreen Nahar

**Affiliations:** 1 Department of Global Health and Infection, Brighton and Sussex Medical School, Brighton, UK; 2 School of Public Health, Addis Ababa University, Addis Ababa, Ethiopia; 3 Department of Development Studies, University of Rwanda, Kigali, Rwanda

**Keywords:** NTDs, Podoconiosis, Disease management, Gender, Lived experience, Structural violence

## Abstract

**Background:**

Podoconiosis is a Neglected Tropical Disease which leads to foot and leg swelling (lymphoedema) and, ultimately, severe disability. People suffering from podoconiosis often cannot continue their economic or social roles for prolonged periods of time and experience stigmatisation. Research points towards women being more affected by podoconiosis and podoconiosis disease management interventions being less effective for women. Women’s participation in a podoconiosis disease management intervention in northern Rwanda was the focus of this study. The benefits women experienced when taking part in the intervention, and the challenges they faced when trying to follow intervention advice were explored.

**Methods:**

This study is based on a focused ethnography which applied several qualitative research methods, including in-depth and key informant interviews, focus group discussions, transect walks, and observations. Women’s experiences with participating in a disease management intervention for podoconiosis in northern Rwanda were analysed through the lens of structural violence.

**Results:**

Our findings demonstrate that while the disease management intervention provided significant benefits to women suffering from podoconiosis, five, often intersecting, contextual domains made it difficult for them to participate optimally. These domains were - the cultural construction of podoconiosis, geographical conditions, personal situations, resource challenges, and intervention implementation limitations. Gender emerged as a cross-cutting factor across all these domains. Women’s precarious lives and power imbalances between the women and the intervention provider impacted women’s ability to optimally engage with the disease management intervention for podoconiosis.

**Conclusions:**

The context of women’s lives crucially impacted their ability to participate in a podoconiosis disease management intervention as intended. The differing needs and challenges of women affected by podoconiosis should be considered when designing and delivering health interventions to achieve more equitable health outcomes.

## Background

The global burden of the Neglected Tropical Disease (NTD) podoconiosis is still not completely understood. However, around 32 countries are either suspected or known to be endemic, and it is estimated that 4 million people suffer from the disease globally [1]. Podoconiosis is caused by the interplay of genetic susceptibility and prolonged barefoot exposure to irritant soil of volcanic origin. Mineral particles in the soil pass through the skin, causing inflammation and fibrosis which in turn block lymphatic drainage. This blockage leads to foot and leg swelling and in the long term to non-filarial lymphoedema and severe disability. In progressed stages, afflicted individuals suffer from intermittent acute inflammatory episodes, often referred to as acute attacks, which intensify pain and disability. Once contracted and not immediately attended to, podoconiosis becomes a chronic disease causing long-term morbidity [2, 3]. People suffering from podoconiosis often cannot continue their economic or social roles for prolonged periods of time and experience stigmatisation [4]. People who have podoconiosis report experiencing stigma due to the bad smell emitted from their legs and the swelling of their feet and legs [5, 6], due to being perceived as bewitched [7] or due to the popular belief that podoconiosis is contagious [5, 8, 9]. They are often avoided or excluded from family, community, and religious activities due to this [5–7].

The development of podoconiosis can be prevented by avoiding contact with the irritant soil through consistent footwear use, foot hygiene and floor coverings [10]. Health interventions for people already suffering from podoconiosis mainly consist of disease management including hygiene and skin care measures to prevent acute attacks [11], proper wound care, bandaging, exercise, elevation of the affected limb, and proper footwear [12].

In Rwanda, the site of this research project, podoconiosis is present in all 30 districts with over 6000 people affected [13]. Evidence shows that podoconiosis affects women more than men in Rwanda [14], and that disease management interventions for podoconiosis are less effective for women [11], however not much is known about why this difference between the two genders exists. Research set in Rwanda found women being disadvantaged in accessing and using protective footwear for farming activities. This was due to difference in decision-making and purchasing power between men and women, and the needs of men and the family being prioritised. Additionally, there existed limited shoe options for women at markets, while aesthetically pleasing shoes also played a role in women’s shoe-wearing habits. Women were often made fun of or faced rumours and backlash from their families and communities when wearing boots [15]. Furthermore, women in Rwanda tend to spend a lot of time on their feet fulfilling gender-based role expectations, which might be a reason for higher podoconiosis incidence rates as well. Next to being responsible for child rearing and tasks within the homestead, women are usually expected to cultivate fields and to fetch water, firewood, and fodder for livestock [16, 17]. According to a report published by the National Institute of Statistics of Rwanda [18], women tend to spend around ten hours more per week on unpaid work compared to men in Rwanda. Gender also seems to enforce the experience of stigma and leads to severe social consequences for women who have podoconiosis in Rwanda. Women’s physical appearance is deemed important for social acceptance and marriageability, while contributing to the economic development of their families is often a central expectation of women in Rwanda. Young women affected by podoconiosis in Rwanda struggle finding a husband and married women who cannot contribute to their families’ economic development due to their diminished physical strength experience a reduction of social status [5, 19]. Women suffering from podoconiosis in Ethiopia reported experiencing intimate partner violence due to their condition which, among others, included their male partners restricting their access to healthcare [20].

These dynamics highlight that podoconiosis is not only a biomedical condition but also a manifestation of broader gender inequities in health, as documented in studies of stigma, disability, and neglected tropical diseases [21–23]. Addressing this burden, therefore, requires gender-responsive health policies and interventions that challenge structural inequalities, promote equitable access to prevention and care, and actively engage with the social norms that marginalise women [24].

Research focusing particularly on the lived experiences of women who have podoconiosis in Rwanda (so far only Igihozo et al. [15] and Bikorimana et al. [5, 19] in Rwanda) and women’s participation in podoconiosis disease management interventions has been under-explored. It was thus crucial to better understand women’s participation in a podoconiosis disease management intervention in Rwanda. Qualitative research on interventions for NTDs, namely mass drug administration [25–31] and disease management [32, 33], has uncovered the importance of centring on the lived experiences of the people interventions are designed for when wanting to achieve desired health outcomes and thus improve health equity.

Structural forces particularly tend to diminish people’s ability to access and optimally engage with healthcare [34, 35], commonly referred to as *structural violence.* Structural violence is a concept used by social scientists to convey how social structures and institutions, including economic, political, legal, religious, and cultural ones, systematically hinder individuals from meeting their needs and achieving their full potential by restricting their agency and opportunities. This is frequently perpetuated through policy, legislation, and resource allocation. Through these devices, structural violence often becomes normalised and almost invisible within a society (see for example: [36–40]). Structural violence within a health and well-being context causes individuals to experience higher disease susceptibility and ill health, as well as limited or no treatment and recovery options. Affected individuals often do not have the possibility to change their circumstances [34]. While the impact of gender-based structural forces on women’s lived experiences with NTDs has been discussed (see, for example: [23, 41, 42]), the framework of structural violence has not yet been used to analyse women’s lived experiences with NTDs.

This study adopted an exploratory, holistic, anthropological approach to uncover contextual aspects that influenced women’s continued participation in a disease management intervention for podoconiosis in Rwanda. This approach enabled the capture of human behaviour and life experiences through qualitative research, aiming to understand interactions between individuals and broader structural forces [43]. The data that emerged were analysed through a structural violence lens.

Before outlining the objectives of this study, it is worth noting that this research was conducted as part of a broader project titled *Social Science for Severely Stigmatising Skin Diseases (5 S) Foundation* [44]. This project focused on strengthening social science capacity around three neglected tropical diseases, podoconiosis, mycetoma, and scabies, across three East African countries: Ethiopia, Sudan, and Rwanda.

### Objectives of the study

The study aims to examine women’s participation in a podoconiosis disease management intervention and to analyse the intervention components through the lens of structural violence. The specific objectives are,

To uncover the contextual challenges affecting women’s participation in podoconiosis disease management interventions.

To inform and improve new or existing podoconiosis disease management interventions by highlighting these challenges.

To promote health equity for women suffering from podoconiosis.

## Methods

### Study design

This study is based on a focused ethnography which applied several qualitative research methods, including in-depth and key informant interviews, focus group discussions, transect walks, and observations. Women’s experiences with participating in a disease management intervention for podoconiosis in northern Rwanda were analysed through a structural violence lens.

We employed focused ethnography since it is an applied research method suited to studies addressing specific research questions within well-defined contexts. This approach involves short-term but intensive fieldwork, with a high demand on both data collection and researcher time [45]. Focused ethnography provides systematic ways to collect and interpret data on topics of practical relevance, with the ultimate goal of improving care and care processes, making it particularly valuable for healthcare research such as ours [46]. Qualitative research typically involves the use of multiple methods for triangulation and validity, for eliciting different types of knowledge and data, and for iterative refinement [47, 48].

We obtained ethical approval from the Research Governance and Ethics Committee of Brighton and Sussex Medical School (RGEC) and from the University of Rwanda Ethics Clearance Committee prior to commencing the study, see Ethics Declaration section further below for more details. We consciously chose not to disclose the exact location of the disease management intervention nor the name of the non-governmental organisation and to use pseudonyms for study participants to protect research relationships and community trust.

### Setting

Fieldwork was conducted in the Northern Province of Rwanda for a total period of 5 months divided into three blocks between September 2022 and May 2023. By conducting fieldwork in blocks during different metrological seasons, the impact of seasonality on study participants’ participation in the disease management intervention could be observed. A district in the Northern Province of Rwanda was chosen as the study site since a disease management intervention for podoconiosis offered by a non-governmental organisation (NGO) was taking place at one of the district’s health centres. Over 300 people are affected by podoconiosis in this district [13].

At the time of fieldwork, the aforementioned NGO was the sole provider of a disease management intervention for podoconiosis in Rwanda. The NGO mainly focused on providing basic hygiene measures aimed to reduce superinfections and improve podoconiosis symptoms. Sometimes, more intense treatment such as compression management of the feet and legs was offered. For people living in the district of our research project located in the Northern Province of Rwanda, the NGO offered a weekly disease management intervention for podoconiosis on the premises of a health centre. People suffering from podoconiosis could attend the intervention free of charge and without health insurance. The NGO provided the materials to manage the illness, namely basins, brushes, vinegar/bleach, soap, body lotion, and ointments, while the health centre provided water access and space for the intervention site. The disease management intervention consisted of patients soaking their feet and legs in a vinegar or bleach solution, washing their feet and legs with soap, scrubbing and filing their feet, and applying body lotion and ointments. Patients living too far away to come to the intervention site on a weekly basis, or too frail, came to the intervention site monthly to pick up intervention materials to follow the intervention procedures from home.

### Participant recruitment

#### Overview of methods and participants

**Table 1:** Summary of method, participants, and sample size

Method	Participants	Sample Size
In-depth Interviews	Women suffering from podoconiosis taking part in the intervention (main participants)	21 women
Key Informant Interviews	People working for the NGO providing the disease management intervention	3 people
Focus Group Discussion 1	Peers of the main participants: neighbours, friends, acquaintances, and a family member who do not suffer from podoconiosis	10 women
Focus Group Discussion 2	Women suffering from podoconiosis and taking part in the intervention, but who are not among the main participants	13 women

#### Demographic information of main participants

**Table 2:** Demographic information of main participants

Demographic Categories	Variables	Amount 21 total
Age Groups	24-35 36-45 46-55 56-65 66-75 76-78	4 1 9 1 4 2
Religion	Catholic Anglican Seventh-day Adventist Pentecostal (ADEPR) Jehovah’s Witness	9 5 4 2 1
Marital Status	Single Married Divorced/Separated Widowed	3 10 3 5
Employment Type	Agriculture Teaching No employment	19 1 1
Highest Level of Education	None 1-3 years of education 4-6 years of education 7-9 years of education Completion of primary and secondary school	8 6 3 2 2
Category of Wealth	1 (poorest) 2 (poor) 3 (rich) 4 (richest)	9 7 5 /

Purposive as well as opportunistic sampling was employed to recruit study participants while data saturation and redundancy served to determine sample size. The saturation point was reached once no new insights or themes were gained from the data collected during interviews, focus group discussions, or other qualitative research methods employed (Table 1).

The main participants of this research project were 21 adult women who had podoconiosis and took part in the disease management intervention in focus (Table 2). The women were between 24 and 77 years old at the time of data collection. Ten women were married, five were widowed, three were separated from their children’s fathers, and three were single. Eighteen of the women were mothers, and of these, fourteen were also grandmothers. The women had between one and eleven children. Eight of our main participants never went to school, eleven did not complete their education, and two graduated from school. Nineteen of our main participants were subsistence farmers, and twelve of our main participants worked as day workers on other people’s fields. One of the main participants worked as a teacher, while one did not work at all. Out of the 21 main participants, nine were Catholic, five were Anglican, four women were Seventh-day Adventist, two were from the Pentecostal Church of Rwanda, and one was a Jehovah’s Witness. All the women lived in villages. The women had suffered from podoconiosis for one year to up to three decades at time of data collection. They had been part of the intervention when it first started operating in 2014 or between three months and six years from the time of data collection.

Attention was paid to recruiting main participants from diverse age brackets and socio-economic backgrounds to capture a broader sample of lived experiences. 19 of our main participants were recruited while taking part in the disease management intervention. Three main participants were recruited through an adult family member who came to the disease management intervention site to pick up items on the main participants’ behalf. Main participants took part in in-depth interviews, observations, and transect walks.

Two focus group discussions were conducted. One of the focus group discussions took place with 10 adult women who were peers of the main participants. These women were neighbours, friends, acquaintances, and in one case a family member, of main participants. However, these peers did not suffer from podoconiosis themselves. Special attention was paid to selecting women from age brackets mirroring the main participants. The second focus group discussion conducted included 13 women who had podoconiosis and took part in the disease management intervention, but who were not among the main participants. They were recruited after attending the disease management intervention at the health centre.

Key informant interviews took place with three people working for the NGO providing the disease management intervention. Key informants were identified and purposively selected based on their roles and approached face-to-face, or by phone by the first author.

After introducing the research team and research project and ensuring study participants had understood the scope of the study and all their questions had been addressed, a date and time was arranged to conduct interviews, observations at participants’ homes, transect walks, and the focus group discussions. Study participants taking part in in-depth interviews, key informant interviews, and in the focus group discussions received a token of appreciation in accordance with local standard rates, equivalent to 5000 Rwandan francs (approximately 3 GBP). Transportation costs were reimbursed, and refreshments were provided during interviews and group discussions. When interviews were conducted in participants’ homes, participants were given a small customary gift in line with local cultural practices. Any type of interview and observation exercise was only conducted in locations and during times suggested by study participants to ensure that it was convenient for them, so they always felt comfortable and secure. As podoconiosis is a stigmatised condition, it was crucial to ensure that the privacy of the participants was always protected. Thus, in some instances, interviews had to be stopped or postponed, or the location changed.

### Data collection

#### Data collection team

The data collection was conducted by CT with help of a female Rwandan researcher, VM, fluent in English and Kinyarwanda, the working language of the area. VM would accompany CT during data collection, serve as interpreter, and conduct interviews in Kinyarwanda under the guidance and in the presence of CT.

#### In-depth interviews

CT, GA, and PN developed interview guides together in English, and then discussed these with DU and VM who helped refine them to better fit to the Rwandan context. VM translated the interview guides into Kinyarwanda, which were also back translated into English to ensure accuracy. The in-depth interviews aimed to uncover the women’s experiences with the intervention, their understanding of podoconiosis as well as of the intervention, the benefits and challenges of participating in the intervention, and the ways in which challenges experienced were mitigated by the women. The women were also asked about their recommendations on how to ameliorate the challenges experienced in their everyday lives.

In-depth interviews were conducted with the main participants to better understand the lived experiences of women with podoconiosis in Rwanda and their access to and participation in the disease management intervention. The main participants took part in 1 to 3 in-depth interviews. First interviews usually took place on the premises of the health centre where the disease management intervention was offered, while second and third interviews usually took place at the main participants’ homes.

#### Key informant interviews

All key informant interviews took place in locations deemed suitable to the respective key informant- either in their offices or in cafés.

#### Focus group discussions

The focus group discussions took place in an assembly room at the health centre where the disease management intervention took place, as the health centre was a well-known and respected place the participants often frequented. The assembly room provided a big and sheltered space.

#### Observations and transect walks

Observations took place to supplement the findings of the interviews and the focus group discussions. The disease management intervention was observed on a weekly basis, 15 times in total. Additionally, three of the main participants were followed for between four and six hours at their homes to observe their daily life routines. These three women were selected to cover three different life stages.

Furthermore, transect walks with the main participants from the intervention site to their homes took place to better understand the challenges the participants faced when walking and to explore the community embeddedness and relationships of the women. As the participants of the research project mainly lived in three distinct areas with different topography, namely in an area close to the volcanoes, a lake area, and a hilly area, three transect walks were conducted.

### Data analysis

Audio-recorded data was transcribed verbatim and then translated back into English by two professional transcribers after the transcribers received training by the first author. Transcription and translation of audio-recorded interviews started simultaneously to data collection. Identifiable information was coded using numbers or pseudonyms to protect the anonymity of the study participants. The coded data were analysed thematically by the first author. Thematic analysis allows researchers to identify, analyse and reflect on the data to report in details [49]. The software NVivo 14 was used as tool to help organise and manage the coding and analysis process. All in-depth interviews conducted with the same participant were combined within one file for a better understanding and comparison of responses. Additionally, observation and field notes, as well as notes from transect walks, were also analysed as data.

## Results

The results show that despite the intervention for podoconiosis providing significant benefits to women and them being eager to take part in it, major challenges affected their optimal participation. These major challenges can be summarised under five, often intersecting, contextual domains of which some had a graver impact than others. These domains were - the cultural construction of podoconiosis, geographical conditions, personal situations, resource challenges, and intervention implementation limitations. Women struggled to regularly come to the health centre to either take part in the intervention on-site or to pick up monthly intervention resources due to distance to the site and difficult geographical conditions, illness, and other responsibilities taking precedence. Additionally, they struggled to afford resources such as (clean) water and soap to wash their feet and legs at home daily as suggested by the intervention provider. Women who wanted to self-manage podoconiosis could often not do so due to economic deprivation and lack of knowledge about a required intervention resource, rendering them highly dependent on the disease management provider.

It is important to note that our main participants had not been diagnosed with podoconiosis before joining the podoconiosis disease management intervention. Thus, women’s explanations of podoconiosis symptoms before joining the intervention, which are connected to local illness explanations, will be presented as a short background section. Following this, the benefits of taking part in the podoconiosis disease management intervention will be explored, which include physical and psychological relief as well as stigma reduction. Then, the challenges the women experienced in participating in the intervention, namely the five contextual domains highlighted above, will be highlighted. Finally, women’s attempts at mitigating the challenges experienced will be elaborated on.

### Background: local illness explanations of podoconiosis symptoms

Before diagnosis and learning about podoconiosis, our main participants ascribed suffering from podoconiosis symptoms to being poisoned or to the local illness *inyamaswa.*

Poisoning is a common local illness explanation. Unexpected and inexplicable deaths, inexplicable abdominal pain, prolonged infertility, and diminished libido were mentioned as signs of being poisoned. According to the women, poisoning was caused by “bad people” perceived as witches casting harmful spells on others due to jealousy, for example. These witches could be community or even family members. Podoconiosis symptoms, especially inexplicable, chronically swollen feet and legs, were often perceived as being caused by poisoning in the women’s communities.

Swelling, leg pain, and especially fever attacks, are symptoms that are also often ascribed to the local illness *inyamaswa*. Thus, when the women were experiencing acute fever attacks, they were often told by elders in their families or communities that *inyamaswa* had caused them. When literally translated from Kinyarwanda, *inyamaswa* means animal, but in the case of the illness, the word implies something animalistic and strange that can harm you. When asked, main participants found it difficult to describe. It was perceived as being something inside the body, that “moves within” like a worm. However, they said it was impossible to know what type of worm it was and what it looked like as it was within the body and thus invisible. The women all stressed that *inyamaswa* suddenly and unexpectedly attacked them and immediately induced pain and severe symptoms including shivering due to chills, “whole-body” fever, weakness, swollen and inflamed legs and feet, and enlarged lymph nodes. *Inyamaswa* was not perceived as a permanent illness. While it induced symptoms, it was seen to pass through the leg until it found an exit. The women stressed that the bursting and peeling of skin on the leg or foot was a sign of *inyamaswa* having passed and that they were recovering from the illness. The traditional medicine used to treat *inyamaswa* included burning either a piece of skin of a serval wild cat or an otter - both animals are seen as medicine in traditional Rwandan narratives - and inhaling the smoke, which leads to sneezing, or using a type of mushroom, which like a puff ball, released a dust-like substance. Like the animal skins, the women would burn the mushroom, smell its fumes, and try to apply the fumes to their feet and legs.

### Benefits of engaging with the intervention

Once our main participants joined the podoconiosis disease management intervention, the most notable benefits the women experienced were physical and psychological relief, as well as stigma reduction. The women were all very dedicated and tried their best to follow through with the intervention on a weekly basis, as the benefits improved their quality of life significantly.

#### Physical relief

Taking part in the intervention led to significant pain-relief which helped sleep at night. No other biomedical nor traditional medicine the women had used had ever helped as much as taking part in the intervention. The women’s podoconiosis symptoms such as foot and leg swelling reduced, and they experienced less acute attacks:

I gained my life. I can eat and sleep in peace! Before I came, I used to get severely ill and spend five days in bed, but I no longer experience those crises! I can walk around and pick the vegetables. Coming to the clinic [the health centre] saved me very much! Mujjawimana

Ultimately, the physical symptom relief the women experienced promoted their ability to walk and stand for longer periods of time, thus increasing their ability to work and take part in social life again.

#### Psychological relief

The intervention also provided psychological relief to the women. Several women highlighted that meeting others with podoconiosis helped them feel less lonely and depressed and that attending the intervention at the health centre gave them a reason to leave home to “breathe fresh air”:

When I’m with others who have the same illness as me, I feel like there is no need to worry. You understand that we all suffer from illness, no one blames the other, no one speaks bad of the other, we know that we are afflicted people. It helps me endure the illness. It helps me not to feel lonely. Akaliza

#### Stigma reduction

The more severe the symptoms the women suffered from, the more they experienced stigma and avoidance by community members. Symptoms such as open wounds and especially bad smell were often perceived to be resulting from poor hygiene by both the women affected and their communities. Smelling bad had a gendered impact, as it was perceived worse when it affected women. This was due to women already being perceived as unhygienic and emitting a bad smell during their menstruation, especially if they did not have the resources for their hygiene needs. Thus, the challenges of being affected by podoconiosis as a woman and menstruating were often compounded. To avoid the stigma associated with being perceived as unhygienic, it was extremely important to the women who had podoconiosis to try as best as they could to present themselves as hygienic and clean. Once the women started taking part in the intervention, the reduction and diminishing of wounds and bad smells had the ‘side-effect’ of the women being accepted and integrated into their communities again. The intervention therefore indirectly led to the diminishment of stigma. For example, after taking part in the intervention and these symptoms vanished, one of our participants was accepted into her church community again:

I go to church every Sabbath, and they usually brag about me and say: (…) “May no one get angry when she’s sharing her story, do you remember how we stigmatised her, look at her now wearing shoes, and look at her legs now and how we stigmatised her back then when they had wounds all over them.” Some even say, “Leave her alone, watch how God does wonders.” Mutesi

Depending on the severity of the symptoms the women displayed at the point of accessing the intervention, taking part in the intervention early on could also fend off potential future stigmatisation.

Taking part in the intervention also meant that the women had access to intervention materials such as vinegar, ointment, soap, and lotion. Since the women came from destitute backgrounds, these were prized possessions they could not easily afford otherwise. Therefore, having access to the intervention materials, which were not only used for their intended purpose, to manage podoconiosis, but also to maintain general hygiene, provided the women with the opportunity to present themselves as clean and hygienic to avoid or reduce stigma.

### Challenges of engaging with the intervention

Although taking part in the disease management intervention notably reduced the women’s symptoms, it did not eradicate them. The women reported still suffering from reoccurring athlete’s foot, swelling, acute attacks, and pain. Two of the women who had more severe cases still had very swollen feet and either open wounds or feet covered in nodules.

Several, often intersecting, contextual domains seem to have had an influence on women’s ability to optimally follow through with the intervention, thus causing symptoms to reoccur. The five contextual domains that emerged from our research were (1) the cultural construction of podoconiosis, (2) geographical conditions, (3) personal situations, (4) resource challenges, and (5) intervention implementation limitations.

### The cultural construction of podoconiosis

In this section, women’s understanding of podoconiosis aetiology, as learned from the intervention providers, will be uncovered. The three aspects of podoconiosis aetiology, namely heritability, barefoot walking, and soil composition will be explored. Women’s perception of the independence of these three aspects as well as their incorporation of local factors into the illness explanation will be highlighted. Finally, the impact of the women’s perception of podoconiosis aetiology on intervention success will be examined.

While nowadays, health interventions tend to acknowledge the importance of socio-cultural context in their implementation more than in the past [50], ‘cultural barriers’ including local health beliefs are often assumed to be the reason for the failure of biomedical interventions [51, 52]. However, while cultural context does seem to play a role in this study, as the women’s understanding of podoconiosis did not exactly match with the podoconiosis aetiology they were told by the NGO providing the disease management intervention, this did not seem to hinder women’s participation in the intervention, as the following paragraphs will demonstrate.

#### Heritability

All the women stated they had learned that podoconiosis was heritable, 15 of the 21 women had family members suffering from the same condition. Three of the women stated that podoconiosis being heritable meant it was related to blood, while one of them thought podoconiosis had “something to do with blood type affecting people”.

However, there was some confusion about podoconiosis being heritable. One of the women who did not have family members with podoconiosis questioned where she had acquired podoconiosis from, as the heritable aspect did not fit her case. Another woman had a mother and a husband who also suffered from podoconiosis symptoms before they passed away. As the woman’s husband could have not inherited podoconiosis from her family, she concluded that she transmitted podoconiosis to him by not wearing shoes.

#### Walking barefoot

Walking barefoot was another important factor the women recalled being related to podoconiosis aetiology. All the women but one stated that wearing shoes could prevent podoconiosis, as walking barefoot was one of its root causes. The women who had children and grandchildren would try to ensure their offspring wore shoes to avoid “infection” with podoconiosis. However, interestingly, five women connected acquiring podoconiosis not only with walking barefoot but especially with coming into contact with stagnant and/or dirty water and rain. This might be due to ‘message crossover’ (a term borrowed from Nichter [53], either from the intervention itself, as the women were taught to dry their feet after soaking and to not share their basins and thus the water in it, or from strong public health messaging about the health dangers of stagnant and dirty water sources. As the women also learned that hygiene was important in the healing process, they believed poor hygiene to be another factor causing podoconiosis.

#### Soil composition

Only four women related mineral particles in the soil to podoconiosis, however all four referred to these particles as insects or parasites that live in the soil. Two of the women stated the insect was called “mineral particles” and “silicosis”, respectively. The insect/parasite was described to ‘bite’ or ‘infect’ someone, or to have found a chance to get into the body, while walking barefoot over soil, in lakes, or in the forest. Two of the women recalled that they were taught that this insect/parasite could be found predominantly in the Northern Region but did not know why so.

#### Independence of aetiology aspects

It seemed that most of the women did not realise that all three factors of podoconiosis aetiology they learned from the NGO had to be present for them to experience the illness. The three factors were seen as independent disease origins rather than their interplay.

Three of the women distinguished between different origins of podoconiosis. For example, one woman made a distinction between two types of podoconiosis, one being inherited, and one being caused by mineral particle insects:

There are two types [of podoconiosis], one caused by mineral particle insects and another type that is heritable including mine. I personally think it was heritable; I inherited it from my family. But because I also used to walk barefoot, I also think that I was also bitten by those mineral particles. Mine is heritable, I can even scratch them and clearly show you. Mutesi

#### Incorporation of local factors

Women also sometimes added local illness explanations to their understanding of podoconiosis aetiology. One woman stressed that while she believed she had inherited podoconiosis from her parents, they themselves probably did not inherit it but were in fact poisoned.

Most strikingly, all the women incorporated the local illness *inyamaswa*, described further above, into the podoconiosis aetiology presented to them by the NGO, as swelling, leg pain, and especially fever attacks, are symptoms that are also often ascribed to the local illness. After being exposed to the NGO’s explanation of podoconiosis aetiology, the local illness *inyamaswa*, was perceived as a starting point or one of the early symptoms of podoconiosis. Additionally, *inyamaswa* was also perceived to cause the acute attacks the women suffered from:

You can distinguish them [podoconiosis and inyamaswa]. When inyamaswa is about to come, you tremble and have fever. You feel so much pain and that is when you know it is inyamaswa. Mulekatete

Even after joining the intervention, the women would treat acute attacks perceived to be caused by *inyamaswa* the same way they did before joining, namely by using biomedical pain-relief medication or the traditional medicine described above.

Podoconiosis (*imidido*) symptoms, on the other hand, were described as leading to swelling of the feet and leg, wounds, nodules, and painful and itching sensations that were present even without experiencing acute attacks:

For imidido (podoconiosis), it feels itchy and stops when you apply that medicine we use while bathing. Then it stops paining for like three days and on the fourth day, it starts paining you again. It pains you as if a pincher bug is biting you. Mulekatete

However, during a focus group discussion with women who took part in the disease management intervention, it became apparent that there was no consensus on which symptoms signified podoconiosis and which *inyamaswa*, as the following discussion highlights:

Mujjawimana: When you’re getting better [after a fever attack], the skin peels off.

Interviewer: Is that podoconiosis or just inyamaswa?

Mujjawimana: It is podoconiosis.

Mukobwajana: It is inyamaswa.

Niyonyugura: We just experience the symptoms; we can’t tell what it is!

Mukobwajana: It is inyamaswa!

Mujjawimana: It is podoconiosis.

Niyonyugura: It happens inside the body thus we can’t see what’s going on!

The women could not be sure if they experienced podoconiosis or *inyamaswa* symptoms.

#### Impact of knowledge and local illness explanation

Despite having learned about podoconiosis aetiology through the NGO, this did not seem to help clear the ‘obscure’ character of the illness origins and symptoms. Most of the women were still unsure what had caused the disease:

It just happened like that. I mean I walk around meeting people who have the same condition, what could I think is the cause? I cannot know. Muteteli

As highlighted, to acquire podoconiosis, three factors need to be present: a genetic predisposition, specific soil composition, and prolonged barefoot exposure. Being the only members of their families affected by podoconiosis despite the same living conditions made the heritable aspect not very compelling to many women. Plus, most of the people around the women would also walk barefoot over the same soil but did not acquire podoconiosis. Having a genetic predisposition for a specific disease does not necessarily mean that one acquires it, or that ones’ offspring are born with it, even if other factors promoting this are present. Thus, even when one is familiar with biomedical gene theory, there is an unexplainable, random element to it. From an intervention point of view, it did not seem crucial for the women to exactly understand biomedical podoconiosis aetiology. The women tried their best to wear shoes and follow through with the disease management advice they had received, as they valued the immense symptom relief the intervention provided. Additionally, using specific *inyamaswa* treatments did not seem to harm the women’s well-being or worsen podoconiosis. There were other, more pressing contextual issues making it difficult for the women to follow through with the intervention, be it at the intervention site or home, which will be explored further below.

### Geographical conditions

This section explores the challenges our main participants experienced in participating in the disease management intervention in relation to the geographical conditions of the area they lived in and in which the intervention took place.

The women could rarely afford transportation to the intervention site. Even if they could, transportation was only available for some parts of the journey as the majority lived in areas not connected by a tarmac road. Thus, the women had to walk either all or part of the way to the intervention site. This took them between 30 min and four hours one way. Walking to the intervention site was challenging:

When I am climbing down, I feel hurt; sometimes I have to sit down and see where it goes. When the pain decreases, I keep going slowly, even though it means arriving late- what matters is that I arrive. Agasaro

The women stated they often arrived late as they had to take breaks or walk slowly. On several occasions, the first author observed women arriving late and then soaking their feet for a much shorter time than the recommended thirty minutes. Sometimes, the women would be too late, as everyone had already left, and the intervention materials were locked up again.

The walk back home after taking part in the intervention was even harder, as the women were already tired from the first journey, as we could observe during transect walks with them. The women often only ate one meal a day and usually did not have any food in the morning. On top of that, walking in the hot sun was exhausting, and when it rained, the women would try to take shelter to avoid getting wet. Walking on the uneven, rocky, and often steep paths and roads became even more difficult and slippery when it was raining. Thus, during the rainy season, the women would sometimes decide to stay at home, or, more tragically, once on their way had to return due to heavy rain.

### Personal situations

The contextual domain of personal situations covers our main participants struggles with participating in the disease management intervention due to other personal priorities taking precedence over participation in the intervention. Co-morbidities as well as participants’ life stage related challenges impacting intervention attendance are also highlighted.

#### Other priorities taking precedence

While the women sometimes skipped attending the intervention at the health centre for social obligations, such as funerals or visiting family members who had given birth, the main reasons for not being able to attend were work-related. For women who were farmers cultivating their own fields, it was easier to join the intervention regularly as they had more autonomy to adjust their work schedules. Some of the older women did not cultivate anymore, thus working did not get in the way of attending. However, most of the women did not have a reliable source of income, as many had no or incomplete education and did not own fields. Sometimes, they were offered part-time jobs on the day of the intervention, mainly to help cultivate or weed another person’s field. This put them in a tough spot as they had to make the decision between tending to their symptoms or earning money to provide food for their families. Some of the women stated they would take the job over attending:

One thing costs the price of another… The pain that is felt in the feet can also radiate to the stomach, that’s why you end up helping yourself like that. Agasaro

Other women stressed they would ‘sacrifice’ their Tuesdays to attend the intervention, allowing them to ease their symptoms, but to miss out on food. Sometimes, the women would manage to attend the intervention on a Tuesday morning, but then once back home, would take up a casual job offer to prevent them “from going to bed with an empty stomach”, undoing the benefits of the intervention. Although standing on their feet for a long time caused a lot of pain and increased swelling, the women often did not have any choice:

I experience pain especially in the night because I spend the day standing on my legs. They got swollen again because I have been spending a lot of time standing lately (…) Yesterday I spent the whole day planting crops. What else can I do! Even today, I was planting. Umukundwa

#### Co-morbidity and podoconiosis symptoms

The women could sometimes not join the intervention at the health centre because of other illnesses. Particularly older women highlighted sometimes feeling too tired and not having the energy to attend on a weekly basis, especially since many of them suffered from comorbidities such as hypertension and diabetes. But it was mostly podoconiosis symptoms that hindered women from coming to the intervention site, even for the women who only attended monthly. The bitter irony was that the women would often be in too much pain to walk to the health centre to attend an intervention designed to relieve this pain, as one of the main participants stated: “If they gave me legs, I would never skip the intervention. It’s true”.

Additionally, and very importantly, even if the women managed to attend the intervention at the health centre, walking back and forth to the intervention site reinforced pain and swelling.

#### The impact of life stage on attendance challenges

The challenges the women faced with attending the intervention at the health centre were intensified according to the life stage they were in. Women who had children who were not in school yet could not leave them at home, as often nobody else could look after them. For mothers of babies and toddlers, walking to the intervention while carrying a child was very challenging:

It breaks me, but I’m patient because you’re sick and you want to get well and you’re trying to go there. It’s hard, but I must put up with it because I am sick, and I want to get better. Ishimwe

Older women worried about becoming frailer with age:

There will be a time when I won’t be able to go there. That’s what worries me. Today God enables me to go there and get the medicine [take part in the intervention]. But what will happen when I won’t be able to go there. I can’t help but think about it. Akaliza

Astonishingly, despite all these difficulties, several women stressed they had only missed attending the intervention at the health centre once or twice while having been enrolled in the intervention for several years.

### Resource challenges

The resource challenges our main participants experienced when wanting to follow through with the intervention advice and procedures is highlighted in this section. These are struggles related to the availability and affordability of footwear, water, food, and intervention materials.

The main challenge the women faced when it came to following through with the intervention advice was the lack of resources, namely appropriate footwear, water, and intervention materials needed to manage the illness outside of the intervention space. As highlighted further above, according to the NGO, the patients attending the intervention at the health centre weekly were also supposed to wash their feet and legs with water and soap daily, while the people picking up the intervention materials monthly were supposed to soak their feet and legs in vinegar-solution twice a week as well as to wash their feet and legs with soap and water daily.

#### Availability of footwear

The women had learned the importance of wearing shoes from the NGO. They knew that being barefoot aggravated their symptoms and thus possessed shoes. The NGO recommended wearing boots or closed shoes, yet, in the beginning, the women’s feet and legs were often still very swollen or had wounds. Thus, wearing closed shoes, and especially boots, was often very painful until the symptoms decreased.

The women would try to buy footwear that was more comfortable in their local markets but simply could not find well-fitting shoes. Some women would thus resort to buying shoes from the men’s section. Still, feeling uncomfortable and in pain due to wearing inappropriate shoes made the women take them off when back home or within their compounds, as the first author could observe in several instances. Most of the women struggled to afford shoes which offered good support and were made of durable materials. On top of that, all the homes of the women visited had uncovered floors, meaning that the women were directly exposed to the irritant soil inside their houses when walking barefoot. In some instances, the first and third author witnessed women walking barefoot in their homesteads.

The issue of affordability of shoes was also compounded by gender. All but one of the women were subsistence farmers. While cultivating and tending to livestock were seen to require wearing boots, and to be men’s tasks, weeding was often the task of women, which required working barefoot to not accidentally trample delicate crops or harden the soil. Therefore, if the women’s families managed to afford a pair of boots, the boots would usually go to the male head of the family.

#### Availability of water

When taking part in the intervention at the health centre, water was always available from one of the water tanks collecting rainwater on the premises. At home, the situation was very different. Some women owned water tanks to collect rainwater which were donated by non-governmental organisations in the region. Others, however, could not afford to buy a water tank and did not receive a donated one either. In this case, the women had to use jerricans, basins, and pans to collect water. Women’s capacity to collect and store water depended on their household’s income and thus the number of vessels that could be used for this purpose:

We have a gutter, but we don’t have enough containers to collect the rainwater in, it’s just two or three jerricans, how long would those last? That would last one day. Mulekatete

Furthermore, the amount of rainwater the women could collect depended on the season. While collecting rainwater and water from natural sources, such as lakes and rivers, was free, the women had to pay for water from public water sources or from community members who owned rainwater tanks. The prices for the water collected from public water sources or community members varied depending on availability and seasonality as well. The women would usually pay between 10 and 30 Rwandan francs (0,006 − 0,02 GBP) for a standard-sized jerrican of 20 L. Sometimes, the women could not afford to buy water. On top of that, public water sources were sometimes located further away from home. Frequently, the closest public water source was not working, under construction, or damaged. If the women could not find a family member or neighbour to fetch water for them, they would have to walk to the water sites themselves and carry the heavy jerricans full of water back home, which was painful and exhausting, often taking between 30 min and one hour one-way.

The women who soaked from home were recommended to use 10 L of water, half a standard-sized jerrican. Often, even if the women’s households possessed some water, other, more pressing needs such as food preparation, laundry washing, or animal watering, were prioritised over soaking feet and legs and even just washing regularly with soap:

Consequences happen but you learn to be patient with them, sometimes you go to bed without taking a shower, and sometimes you get one jerrycan and refuse to overuse it. That way, consequences happen. Mulekatete

On top of that, any water the women collected had to be disinfected for it to be safe for consumption and use. As many women could not afford to buy disinfection agents, the only option they had was to boil water. This in turn required firewood and matches, which the women often could not afford in enough quantity to justify boiling large amounts of water. Thus, they would sometimes use uncleaned water to wash their feet and legs or to soak in the vinegar-solution.

#### Availability of food

The women struggled with having enough food, often only eating once a day in the evening, or having to skip a day during bad times, which often coincided with the seasonality of agriculture, as between harvests, there was little to eat from their own plots or money from selling produce. Sometimes, main participants would resort to cooking and eating the leaves of their bean plants, as the first author could observe. This food poverty was due to not owning or owning only very small plots of land. Jobs as day labourers on other people’s fields were also dependent on the season. Having podoconiosis itself impacted women’s ability to farm further exacerbating the situation. Thus, when asked what their challenges were and what would help them in relation to the intervention, the majority spoke about food:

They availed the medication for me and if they can also help us by maybe giving us food… would you bathe if you don’t eat? … Everything revolves around eating! What would you be able to do if you’re hungry? Would you be able to cultivate if you haven’t eaten? You would dig without any strength. … Who has ever gotten sick when they had food? Uwamahoro

While the women were grateful for the intervention helping reduce their symptoms, the root cause of their challenges, poverty, and acute challenges resulting from it, namely hunger, were not addressed and sometimes not even acknowledged by the NGO staff, despite being told about it.

#### Intervention materials

Women who were supposed to follow the intervention from home reported not receiving enough intervention materials, namely vinegar, soap, and body lotion, to last the four weeks. The soap and lotion would run out before the end of the month. This was due to both being used for maintaining overall hygiene and not just managing podoconiosis. It seemed as if all the women, be it the ones receiving soap fortnightly or monthly, used the soap to manage the illness and maintaining hygiene respectively.

This was compounded by the fact that most of the women had children and grandchildren. The women reported sharing the soap they received from the NGO with their dependents, as the women and their families often could not afford it.

### Intervention implementation limitations

This final section on the challenges our main participants experienced when taking part in the disease management intervention covers the implementation limitations of the intervention. These include stigma related to the location of the intervention, women’s limited knowledge on the use of vinegar in the intervention procedures, as well power imbalances between the NGO staff and people taking part in the disease management intervention.

#### Location and stigma

The health centre in which the disease management intervention took place is very well known and trusted among the population it serves. However, when leprosy was still endemic in Rwanda, nuns working at the health centre would look after people suffering from leprosy abandoned by their families. Although this took place several decades ago, it remained in the adjacent communities’ memory, as the following quote from a peer of the main participants who took part in the peer focus group discussion demonstrates:

We used to see them when we went to the mass back when we were young. We saw them at the mass whenever they brought them. We could see that they had swollen lumps. They sat alone; no one was allowed to get near them. We only saw them [at the health centre], they were called lepers.

The communities of the women who had podoconiosis would often assume the women had leprosy when they displayed wounds and nodules and thus would avoid them, most probably due to this reason. Thus, while the health centre itself was very reputable in the region, the disease management intervention for podoconiosis taking place on its premises might have stimulated or maintained some of the community stigma surrounding podoconiosis being identified as leprosy, which thus created fear of contagion.

#### Knowledge of vinegar

The main participants always refered to the vinegar used for soaking as “medicine”, which seems to have two explanations. On one hand, soap and lotion were common hygiene products the women were using outside of the intervention as well. However, in Rwanda, vinegar was not a common household item:

They explained it to us and told us its name. I think the medicine can be used to prepare salad too [laughing]. I think we use it to maybe to get those parasites out of our feet [laughing] as it removes parasites from vegetables during salad preparation. Isimbi

The women used vinegar in a health context when tending to their illness. Vinegar, next to anti-fungal ointment, was the healing agent in the disease management intervention. Thus, it made sense to call vinegar medicine, as this was its purpose in this context.

On the other hand, it is important to note that only five women knew that the medicine they referred to was in fact vinegar. Out of these five, three women were literate, thus able to read what was written on the bottle, and one was the president of the patients’ committee who was specifically knowledgeable of the intervention. All other women, including the women who took part in the focus group discussion, stated that they did not know the name of the “liquid medicine”. The women being illiterate and thus not able to read what is written on the bottle seemed to play a big part in that.

Furthermore, as the NGO staff stated, they did not encourage patients to buy and use vinegar for soaking at home, this was only seen as a last resort for patients who could not regularly attend the intervention at the health centre. So, while the NGO staff might have mentioned that they were using vinegar during the intervention procedures, they did not make a specific effort to educate patients on this. Even if women might have heard from the NGO that they were using vinegar at some point, the women had not bought it or used it outside of the intervention before, thus they might have had difficulties recalling the name. Additionally, some of the women stated they had only seen this type of ‘medicine’ at the intervention site. This meant that the women, besides the five who knew it was vinegar, could not buy vinegar to use to self-manage, meaning soak their feet and legs at home, despite it being easily available at markets.

While the women might not have found it very compelling to believe that a household product could possess the same healing capabilities as biomedical medicine, the following statement underlines that the women did not in fact know they were using vinegar. Instead, they thought they were using ‘medicine’ related to the biomedical sphere which therefore was only available at health facilities:

Regarding that challenge that we’re talking about, when a person faces it and can’t come for treatment or can’t come to soak, the disease worsens. Thus, as my colleagues said, if you could distribute the medication to nearby health posts it would help a person living nearby to access the medication. She could access the medication without any hardships/difficulties.

As the last quote demonstrates, women who attended the intervention on a weekly basis stressed wanting to be able to access this ‘medicine’ outside of the intervention space as well, to be more independent. As highlighted, geographical conditions and their personal situations could hinder women from attending the intervention weekly. Therefore, if women skipped attending the intervention, they had to wait to follow through with the disease management intervention until the next Tuesday they could attend again.

Standard podoconiosis treatment protocol according to the NGO recommends soaking feet and legs in vinegar-solution twice a week. However, only the patients who would pick up the intervention materials monthly were told to soak twice a week and given the materials to do so. Patients who attended the intervention on-site were limited to soaking their feet and legs once a week. According to the NGO, this was due to the patients’ wishes, as it was too difficult for them to come to the intervention site twice a week due to geographical conditions and their personal situations. However, resource constraints of the NGO and limited staff availability might play a role in this as well. The women even stated that ideally, they would like to soak their feet and legs again around three days after taking part in the intervention at the health centre, as the pain would then reoccur, underlining the fact that soaking twice a week would be ideal.

#### Power imbalances

Not knowing the specific ins and outs of the intervention, such as which ‘medicine’ they were using, seems to be related to the traditional, hierarchical doctor-patient relationship still very present in Rwanda, see for example [54–56]. The NGO staff were perceived to be doctors by the women, as they looked after their health.

Several women stated they did not ask about the ‘medicine’, as though they did not think it was their place to do so, which was most probably reinforced by their illiteracy and lower social status. Doctors, having the power to heal, were often equated to God:

I don’t know, how would I know. We only come here for treatment, and they explain to us what kind of disease we are suffering from, and we would cooperate with God and heal. None knows the disease that is in their body, the son of man would know it? Only God knows, and the doctors. Because (…) doctors are also the same as God because they know what cures a person and what heals a person. Garuka

Even if the women addressed the NGO staff, their requests were usually denied, leaving them no choice but to accept the situation. Several women noted that they had asked the NGO staff to be allowed to receive intervention materials monthly. However, the request was granted for only one woman , but only after the intervention of the president of the patients’ committee:

We work together because we do not have another choice. A lot of people have asked to receive medicine from home, but they didn’t get it. Akaliza

The women had very little independence or say when it came to deciding how and when to follow through with the disease management intervention. While the women who attended the intervention weekly basically already self-managed their illness at the intervention site, they were denied doing so at home. While the main reason for this, according to the NGO staff, was the worry of patients overusing vinegar when unsupervised, the women were very much aware of the specific amount of bottle caps of ‘medicine’ (vinegar) they needed to add to the water. Furthermore, the NGO staff did allow people who could not attend the intervention on site weekly to self-manage, namely soaking their feet and legs in a vinegar-solution unsupervised at home. The argument about mismanagement does not seem that strong and also made patients who had to attend the intervention on-site weekly highly dependent on the NGO. This created a two-class system which unintentionally provided more self-determination to patients attending the intervention monthly, and to literate patients able to read what was written on the ‘medicine’ (vinegar) bottles who also tended to be economically better off and thus have the option to procure the vinegar themselves and to self-manage at home, as was the case for one of the women who was a teacher.

### Mitigation attempts for intervention challenges

Despite the challenging situation our main participants found themselves in, they tried to find ways to make ends meet and follow through with the intervention advice. This section highlights the ways in which the women tried to mitigate the impact of not being able to attend the disease management intervention as well as of not being able to afford and access resources required for disease management. Women’s attempts of mitigating the implementation limitations of the intervention are also examined.

#### Mitigation attempts for non-attendance

The women tried to plan ahead. Sometimes, if there were enough materials in stock, the women who attended the intervention weekly were able to take home some leftover vinegar and lotion in small plastic bottles and containers they brought with them to use in case they had to skip attendance at the intervention site.

If the women could not attend the weekly intervention at the health centre or come to the intervention site to pick up the monthly intervention materials, they would sometimes ask another patient who lived close by to bring them some leftover lotion or vinegar or send relatives to pick up the materials for them, respectively.

#### Mitigation attempts for resource challenges

##### Adequate Shoes

Women who could not fit into standard shoes tied pieces of cloth or banana leaves around their feet and legs to protect them. Women who could fit into shoes could often not afford sturdy footwear, so instead of not wearing any shoes, they resorted to buying cheaper plastic slippers or closed plastic shoes in the shape of ballet flats. However, these plastic shoes broke easily while walking over the stony terrain. On top of that, the plastic ballet flat-type shoes cut into the women’s swollen feet and heels as they were quite narrow and stiff, causing more pain and additional wounds, while the plastic slippers did not offer any support when ascending or descending the hills the women were surrounded by. Additionally, neither type of shoe protected the women from the dusty and dirty roads, so they had to clean their feet again once they reached home after taking part in the intervention.

##### Water

The women could sometimes collect water from one of their neighbours who owned rainwater tanks. This meant shorter walking distances and was sometimes, as a neighbourly gesture, free of charge, as the first author could observe during a home visit.

The women tried to use any amount of water they could afford to use for washing and soaking, even if it did not fully cover their feet up until their ankles when soaking:

I take one small jerrican to use instead of two which are the standard to be used. Thus, instead of the standard thirty minutes, I add an extra five minutes to spread water to other parts which were not covered while soaking. Agasaro

If the women could not access water at all, they would not wash nor soak their feet and legs in the evening, worsening their condition and impacting their sleep.

##### Intervention materials

If the women had spare money, they would try to buy soap and lotion to use at home. As the NGO-provided body lotion was often too expensive to afford, the women used petroleum jelly instead, which cost around one hundred Rwandan francs (0.06 GPB). Some women highlighted that using petroleum jelly was not ideal, as it made dirt stick to the feet and legs, but they often had no other choice. If the women who soaked their feet and legs from home could not buy materials that had run out before the next monthly pick-up, they would just use whatever materials they had at their disposal and omit the missing ones:

I soak twice [a week] but apply the lotion once a week! It is all about getting used to it! There’s a saying: ‘He who doesn’t have means behaves humbly!’ Would I apply the lotion every time I soak and so on, hoping to get the lotion from somewhere? A person trains him/herself and gets used to it! Uwamahoro

#### Mitigation attempts for intervention implementation limitations

As a consequence of not being able to self-manage podoconiosis at home due to a lack of knowledge about vinegar, the women used alternative medicine to tend to pain which reoccurred a few days after taking part in the intervention on site. They boiled the leaves of plants, such as *igisugisugi* (*Senecio hadiensis*), *umuravumba* (*Tetradenia Riparia*) and *umuhoko* (*Phytolacca dodecandra*), put them on their feet and legs for pain relief, or soak their feet in water with the leaves as well as black salt and rosemary. According to one woman, they also ground specific herbs, mixed them with ghee, and applied them on their feet. This eased the pain and helped them sleep. The women supported each other with procuring the herbs and ghee, as some lived in areas where the specific herbs grew or produced ghee. One of the women had a neighbour who was a traditional healer who gave her medication to use for free as a trial to see if it would ease her pain. The medication did help but only provided pain-relief for one day. Still, she combined going to the intervention and using the medication her neighbour gave her. Another woman applied clay she bought from a traditional healer, washed it off, and then applied ghee to her body, which helped her feel better and relaxed.

As well as employing traditional healing, the women consulted public biomedical health facilities close to their homes for pain relief, especially when experiencing acute attacks. Nevertheless, all the women who took part in this research study stressed that biomedical healthcare pain-relief was not as efficient as soaking their feet and legs in the vinegar-solution.

Several women stated also relying on intercessor groups, organised groups commonly found within Christian denominations who pray for a faith member’s healing and recovery from illness, as the women hoped this would help them get cured in the future:

There’s no way I would ignore that … I still have hope to probably get better someday. Because with God, nothing is impossible thus I’ll get cured, and this won’t be the cause of my death. Mulekatete

## Discussion

While the women experienced symptom relief from participating in the intervention, the intersection of several contextual domains hindered them from fully experiencing its benefits. The contextual domain of the cultural construction of podoconiosis did not seem to have as big as an impact on women’s participation in the disease management intervention, as women very much understood its benefits and tried their best to follow through. Women struggled to regularly come to the health centre to either take part in the intervention on-site or to pick up monthly intervention resources mainly due to structural challenges. Although women tried their best to mitigate these challenges, they were too big to fully overcome. Research on podoconiosis in Ethiopia has also highlighted the impact structural forces have on people’s self-management of the illness and participation in podoconiosis interventions. Similar to our findings, running water, electricity, and paved roads were often not available in the communities of people affected by podoconiosis [57]. Accessing water was dependent on seasonality and income [8], with immediate water needs such as for drinking and cooking being prioritised over washing [8, 58], while available water sources were often of poor quality [22]. Additionally, collecting water was challenging for people suffering from podoconiosis due to difficulties walking [8] and long distances to water sites [58]. Challenges surrounding shoe wearing due to access to suitable and sturdy shoes in rural areas [59] and thus resorting to unprotective footwear [58], financial inaccessibility and unsuitability for livelihood practices such as farming [60], were also highlighted. Research set in Ethiopia [60] and Rwanda [15] also found that women faced greater challenges due to their gender when it came to shoe wearing. Not being able to afford medication such as painkillers to ease acute attacks was also reported by Engdawork et al. [57] in Ethiopia. Access to interventions for podoconiosis in Ethiopia was initially often delayed due to fear of being associated with the illness and consequently being stigmatised [3, 9], as our study also found. Once people with podoconiosis in Ethiopia started to engage with disease management interventions, the long distance between patients’ homes and intervention sites had a negative impact, as patients often had no other option than to walk there [3, 9, 22, 57], worsened even more by heavy rainfall [9]. Long walks exacerbated symptoms which hindered journey completion and future attendance [9]. In line with our findings, Tora et al. [3] and Tsegay et al. [9] found that even if transportation options existed, these were often unaffordable and that especially elder patients suffered from the long distance. The findings of Tsegay et al. [9] on the difficulty of attending podoconiosis interventions due to work obligations and social activities such as attendance of funerals, as well as the findings of Tora et al. [3] on illness preventing patients from attendance corroborate our findings. A study by Adler et al. [61] on women’s health priorities, preferences, and experiences in care in Rwanda further underlines our arguments that women struggled to access care due to transportation barriers and work taking precedence, especially since the economic impact of illness was notable even for women who possessed community-health based insurance. Other research set in Rwanda also found distance to health facilities [62–65] due to limited transportation options [63, 65] especially in mountainous regions [64] and unaffordability of transportation [63, 66, 67] to be challenges in accessing healthcare.

The consequence of some of our study participants not being able to access intervention materials, including vinegar, nor afford intervention materials to self-manage the illness outside of the intervention space made them dependent on the NGO. On top of that, at the time, there existed no other podoconiosis disease management providers in Rwanda, thus if women wanted to tend to their illness, they had to take part in the NGO’s intervention, further entrenching dependency issues. A study by Cubaka et al. [55] on patient insights on patient–provider communication in Rwanda also found patients to be strongly dependent on healthcare providers due to hierarchical, paternalistic relationships. Providers were often perceived as God-like, especially by illiterate patients who trusted and rarely actively questioned decisions. Providers not disclosing information on the type of medication provided was also noted in other studies set in Rwanda. Rwandan patients who dared to speak up were often told they were not in a position to do so, enforcing power imbalances even more [61, 65]. As patients in Rwanda needed to access care for their health issues, they had no other option than to accept this power imbalance and return to healthcare providers even if they were unsatisfied with their services [55], similar to our study participants. Along the same vein, a study on Parkinson’s disease set in Kenya [68] found that healthcare providers tended to provide more information to patients perceived to have a higher educational background as they assumed these patients would be more capable of understanding it.

However, it is also important to keep in mind that in our research study, the NGO was not only the sole provider offering podoconiosis treatment in Rwanda but also offered it free of charge and without requiring community-based health insurance. The NGO was about to scale up its efforts within the country and had new points of focus, thus it was not easy to keep maintaining the intervention taking place at the health centre. Thus, with the sole provider of the disease management intervention for podoconiosis in Rwanda experiencing its own challenges, and the women living precarious lives, it was almost impossible for the women to break out of the vicious cycle they found themselves in. As Farmer [34] argued in his seminal work on the importance of understanding the impact of structural forces on people’s ability to access and engage with healthcare: ‘Throughout the world, those least likely to comply are those least able to comply’. Mattes [35] poignantly stresses that the ‘choices’ patients have in relation to participation in healthcare are often shaped by ‘severe economic scarcity and the mere struggle for survival’.

### Implications for policy and practice

Our study shows that the context of women’s lives crucially impacted their ability to participate in a podoconiosis disease management intervention as intended. We recommend providing access to podoconiosis disease management intervention materials closer to the patients’ homes, for example at health posts found at village level, as a first step. In addition, providing all patients with the knowledge needed and the option to self-manage their condition outside intervention spaces would be a crucial step towards steering away from paternalism and unintended dependency on intervention providers. Overarchingly, the goal should be integration within existing state-sanctioned health services while ensuring the provision of services free of charge, which includes addressing the issue of unaffordability of community-based health insurance. The differing intersectional needs and challenges of women suffering from podoconiosis should be considered when designing and delivering health interventions to achieve more equitable health outcomes. Ideally, patients suffering from podoconiosis should be involved in intervention design, delivery, and evaluation.

### Reflections on gender and power

While research on women suffering from podoconiosis in Ethiopia revealed that women experienced intimate partner violence, including hindered healthcare access [20], our results do not conform to the general patterns of intimate partner violence reported. Below, we provide our interpretation of this finding.

Our main participants did not mention intimate partner violence nor being restricted in their participation in the disease management intervention by male partners. This might be due to several reasons. Eight of our main participants were either widowed, separated, or single, fourteen were already grandmothers, which meant they had fulfilled their gender role expectations and gained more power within their households. Furthermore, most of our main participants had been taking part in the disease management intervention for several years. Their partners could see the benefits of the intervention, which not only included physical improvement leading to better gender role fulfillment of the women but also being able to access soap and gifts such as rice the women received for Christmas. This is something we uncovered in a different component of our bigger research project, in which we also interviewed partners of our main participants. On top of that, the intervention was offered free of charge, and no health insurance was required, which allowed women to be financially independent from their partners in this regard. The location of the intervention being a health centre could have also helped, as it was normal to go to health centres for routine check-ups and vaccinations. Finally, it is important to note that the topic of intimate partner violence is not a topic which is easily discussed or shared. It requires time and longer relationships between researchers and participants for these topics to come up, which we did not have due to conducting a focused ethnography.

## Conclusions

While the disease management intervention provided significant benefits to women suffering from podoconiosis, five, often intersecting, contextual domains made it difficult for them to participate optimally. Our findings demonstrate that the women’s precarious lives and power imbalances between the women and the intervention provider impacted women’s ability to optimally engage with the disease management intervention for podoconiosis.

## Data Availability

Data will be available to use for other researchers via a University of Sussex secure repository.

## Abbreviations

NGO: Non-governmental Organisation
NTD: Neglected Tropical Disease

## References

[1] Deribe K, Cano J, Newport MJ, Pullan RL, Noor AM, Enquselassie F, et al. The global atlas of podoconiosis. Lancet Glob Health. 2017;5(5):e477–9. 28395836 10.1016/S2214-109X(17)30140-7 PMC5390851

[2] Deribe K, Cano J, Trueba ML, Newport MJ, Davey G. Global epidemiology of podoconiosis: A systematic review. PLoS Negl Trop Dis. 2018;12(3):e0006324. 29494642 10.1371/journal.pntd.0006324 PMC5849362

[3] Tora A, Davey G, Tadele G. Factors related to discontinued clinic attendance by patients with podoconiosis in southern Ethiopia: a qualitative study. BMC Public Health. 2012;12(1):902. 23095311 10.1186/1471-2458-12-902 PMC3503806

[4] Chandler DJ, Grijsen ML, Fuller LC. With Bare Feet in the Soil: Podoconiosis, a Neglected Cause of Tropical Lymphoedema. Dermatology. 2021;237(2):236–47. 32101870 10.1159/000506045

[5] Bikorimana JP, Davey G, Mukabera J, Shahaduz Z, Mugume PJ, Nahar P. Uncovering intersecting stigmas experienced by people affected by podoconiosis in Nyamasheke district, Rwanda. PLoS Negl Trop Dis. 2024;18(10):e0012603. 39432535 10.1371/journal.pntd.0012603 PMC11527326

[6] Gebreselassie AF, Shimelash N, Kallon A, Mkondo G, Huston T, Schurer JM. We no longer experience the same pain’: a cross-sectional study assessing the impact of Heart and Sole Africa’s podoconiosis prevention education program. Trans R Soc Trop Med Hyg. 2024;118(8):520–6. 38465481 10.1093/trstmh/trae007

[7] Risat MIK, Davey G, Mugume P, Zaman S. Understanding the caring network of podoconiosis patients in Rwanda: a qualitative study. Front Trop Dis. 2024;5.

[8] Banks HS, Tsegay G, Wubie M, Tamiru A, Davey G, Cooper M. Using Qualitative Methods to Explore Lay Explanatory Models, Health-Seeking Behaviours and Self-Care Practices of Podoconiosis Patients in North-West Ethiopia. PLoS Negl Trop Dis. 2016;10(8):e0004878. 27536772 10.1371/journal.pntd.0004878 PMC4990189

[9] Tsegay G, Wubie M, Degu G, Tamiru A, Cooper M, Davey G. Barriers to access and re-attendance for treatment of podoconiosis: a qualitative study in northern Ethiopia. Int Health. 2014;7(4):285–92. 25540135 10.1093/inthealth/ihu085

[10] Deribe KEW, Tekola-Ayele F, Davey G, Podoconiosis. Endemic Non-filarial Elephantiasis. In: Gyapong JOBB, editor. Neglected Tropical Diseases – sub-Saharan Africa. 1st ed. ed: Springer; 2016. pp. 231–49.

[11] Negussie H, Molla M, Ngari M, Berkley JA, Kivaya E, Njuguna P, et al. Lymphoedema management to prevent acute dermatolymphangioadenitis in podoconiosis in northern Ethiopia (GoLBeT): a pragmatic randomised controlled trial. Lancet Global Health. 2018;6(7):e795–803. 29773516 10.1016/S2214-109X(18)30124-4 PMC6562300

[12] Health FDRoEMo. Lymphatic Filariasis and Podoconiosis Morbidity Management and Disability Prevention Guidelines. 2016.

[13] Deribe K, Mbituyumuremyi A, Cano J, Jean Bosco M, Giorgi E, Ruberanziza E, et al. Geographical distribution and prevalence of podoconiosis in Rwanda: a cross-sectional country-wide survey. Lancet Glob Health. 2019;7(5):e671–80. 30926303 10.1016/S2214-109X(19)30072-5 PMC6465958

[14] Bikorimana JP, Bayisenge U, Huston T, Ruberanziza E, Mbonigaba JB, Dukuzimana MJ, et al. Individual and familial characteristics of patients with podoconiosis attending a clinic in Musanze District, Rwanda: A retrospective study. Trans R Soc Trop Med Hyg. 2020;114(12):947–53. 33169149 10.1093/trstmh/traa068 PMC7738661

[15] Igihozo G, Dusabe L, Uwizeyimana J, Nyiransabimana E, Huston T, Schurer JM. We all think boots are meant for men: A community-based participatory assessment of rural women’s barriers to preventing podoconiosis in Rwanda. PLOS Global Public Health. 2024;4(5):e0002773. 38701034 10.1371/journal.pgph.0002773 PMC11068164

[16] Kvinna till Kvinna Foundation. From gender-relevant to gender-transformative climate finance: Rwanda case study. Stockholm, Sweden; 2024.

[17] Saha A, Carreras M, Chopra D. Unpaid care work: A feminist analysis of time diaries from Ghana. London: Bangladesh and Rwanda; 2023.

[18] National Institute of Statistics of Rwanda. National Gender Statistics Report 2024. Rwanda: Kigali; 2024.

[19] Bikorimana JP, Mukabera J, Davey G, Mugume PJ, Nahar P. Individual identities and stigma inequalities: insights from the experience of people affected by podoconiosis in Rwanda. Int J Equity Health. 2025;24(1):254. 41057841 10.1186/s12939-025-02638-5 PMC12506170

[20] Tsegay G, Deribe K, Deyessa N, Addissie A, Davey G, Cooper M, et al. I should not feed such a weak woman’. Intimate partner violence among women living with podoconiosis: A qualitative study in northern Ethiopia. PLoS ONE. 2018;13(12):e0207571. 30521548 10.1371/journal.pone.0207571 PMC6283553

[21] Ayode D, Tora A, Farrell D, Tadele G, Davey G, McBride CM. Dual perspectives on stigma: reports of experienced and enacted stigma by those affected and unaffected by podoconiosis. J Public Health Res. 2016;5(2):jphr.2016.689. 10.4081/jphr.2016.689 PMC5062755 27747202

[22] Tomczyk S, Tamiru A, Davey G. Addressing the Neglected Tropical Disease Podoconiosis in Northern Ethiopia: Lessons Learned from a New Community Podoconiosis Program. PLoS Negl Trop Dis. 2012;6(3):e1560. 22428078 10.1371/journal.pntd.0001560 PMC3302806

[23] Wharton-Smith A, Rassi C, Batisso E, Ortu G, King R, Endriyas M, et al. Gender-related factors affecting health seeking for neglected tropical diseases: findings from a qualitative study in Ethiopia. PLoS Negl Trop Dis. 2019;13(12):e0007840. 31830026 10.1371/journal.pntd.0007840 PMC6907747

[24] World Health Organization. Gender and health2022 15.01.2026. Available from: https://www.who.int/health-topics/gender

[25] Arjyal A, Parajuli A, Kharel C, Del Barrio MO, Baral SC. Understanding gender and its intersection with social stratifiers on prevention and care seeking behavior of lymphatic filariasis in Nepal. Infect Dis Poverty. 2023;12(1):77. 37608332 10.1186/s40249-023-01126-8 PMC10463999

[26] Bardosh K, Inthavong P, Xayaheuang S, Okello AL. Controlling parasites, understanding practices: the biosocial complexity of a One Health intervention for neglected zoonotic helminths in northern Lao PDR. Soc Sci Med. 2014;120:215–23. 25261615 10.1016/j.socscimed.2014.09.030

[27] Dalisay SNM, Belizario VY, Joe JAS, Lumangaya CR, Cruz RD. Critical medical ecology and intersectionality perspectives in schistosomiasis prevention and control in selected communities in Mindanao, the Philippines. J Biosoc Sci. 2023;55(2):306–25. 35022107 10.1017/S0021932021000766

[28] Hastings J. Rumours, riots and the rejection of mass drug administration for the treatment of schistosomiasis in Morogoro, Tanzania. J Biosoc Sci. 2016;48(Suppl 1):S16–39. 27428064 10.1017/S0021932016000018

[29] Parker M, Allen T. Does mass drug administration for the integrated treatment of neglected tropical diseases really work? Assessing evidence for the control of schistosomiasis and soil-transmitted helminths in Uganda. Health Res Policy Syst. 2011;9:3. 21211001 10.1186/1478-4505-9-3 PMC3024987

[30] Parker M, Allen T, Hastings J. Resisting control of neglected tropical diseases: dilemmas in the mass treatment of schistosomiasis and soil-transmitted helminths in north-west Uganda. J Biosoc Sci. 2008;40(2):161–81. 17761005 10.1017/S0021932007002301

[31] Pearson G. Low prevalence of intestinal schistosomiasis among fisherfolk living along the river Nile in north-western Uganda: a biosocial investigation. J Biosoc Sci. 2016;48(S1):S74–91. 27428067 10.1017/S0021932016000237

[32] Dean L. Narratives of Neglect: Privileging the priorities of affected persons to support health systems strengthening and the development of equitable people-centred responses to Neglected Tropical Diseases. in Liberia: University of Liverpool; 2020.

[33] Dean L, Tolhurst R, Nallo G, Kollie K, Bettee A, Theobald S. Neglected tropical disease as a ‘biographical disruption’: Listening to the narratives of affected persons to develop integrated people centred care in Liberia. PLoS Negl Trop Dis. 2019;13(9):e0007710. 31490931 10.1371/journal.pntd.0007710 PMC6750611

[34] Farmer P. Infections and Inequalities: The Modern Plagues. 1 ed. University of California Press; 1999.

[35] Mattes D. We Are Just Supposed to Be Quiet: The Production of Adherence to Antiretroviral Treatment in Urban Tanzania. Med Anthropol. 2011;30(2):158–82. 21400351 10.1080/01459740.2011.552454

[36] Farmer P. An Anthropology of Structural Violence. Curr Anthropol. 2004;45(3):305–25.

[37] Farmer PE, Nizeye B, Stulac S, Keshavjee S. Structural Violence and Clinical Medicine. PLoS Med. 2006;3(10):e449. 17076568 10.1371/journal.pmed.0030449 PMC1621099

[38] Galtung J. Violence, Peace, and Peace Research. J Peace Res. 1969;6(3):167–91.

[39] Gilligan J. Violence: reflections on a national epidemic. New York: Vintage Books; 1996. Available from: http://catalog.hathitrust.org/api/volumes/oclc/35714306.html

[40] Uvin P. Aiding violence: the development enterprise in Rwanda. West Hartford (Conn.): Kumarian; 1998.

[41] Arjyal A, Parajuli A, Kharel C, Del Barrio MO, Baral SC. Understanding gender and its intersection with social stratifiers on prevention and care seeking behavior of lymphatic filariasis in Nepal. Infect Dis Poverty. 2023;12(1):77. 37608332 10.1186/s40249-023-01126-8 PMC10463999

[42] Partnership AD. Discussion Paper: The gender dimensions of neglected tropical diseases. HIV, health and development group bureau for policy and programme support united nations development programme; 2020.

[43] Stellmach D, Beshar I, Bedford J, du Cros P, Stringer B. Anthropology in public health emergencies: what is anthropology good for? BMJ Global Health. 2018;3(2):e000534. 29607097 10.1136/bmjgh-2017-000534 PMC5873540

[44] Zaman S, Nahar P, MacGregor H, Barker T, Bayisenge J, Callow C, et al. Severely stigmatised skin neglected tropical diseases: a protocol for social science engagement. Trans R Soc Trop Med Hyg. 2020;114(12):1013–20. 33324991 10.1093/trstmh/traa141 PMC7738656

[45] Knoblauch H. Focused ethnography. Forum qualitative sozialforschung/Forum: Qualitative Social Res. 2005;6(3).

[46] Higginbottom GMA, Pillay JJ, Boadu NY. Guidance on Performing Focused Ethnographies with an Emphasis on Healthcare Research. Qualitative Rep. 2013;18(9):1–6.

[47] Denzin NK. The Research Act: A Theoretical Introduction to Sociological Methods. New York: Routledge; 2009.

[48] Patton MQ. Qualitative Research & Evaluation Methods. 4th ed. Thousand Oaks, CA: SAGE; 2015.

[49] Braun V, Clarke V. Using thematic analysis in psychology. Qualitative Res Psychol. 2006;3(2):77–101.

[50] Lock MNM. Introduction: From documenting medical pluralism to critical interpretations of globalized health knowledge, policies, and practice. In: Lock MNM, editor. New Horizons in Medical Anthropology: Essays in Honour of Charles Leslie. 1st ed. ed: Routledge; 2002. pp. 1–34.

[51] Chandler CIR. Knowledge, attitudes, and practice surveys. The International Encyclopedia of Anthropology; 2018. pp. 1–2.

[52] Launiala A. How much can a KAP survey tell us about people’s knowledge, attitudes and practices? Some observations from medical anthropology research on malaria in pregnancy in Malawi. Anthropol Matters. 2009;11:1–13.

[53] Nichter M. Global health why cultural perceptions, social representations, and biopolitics matter. Tucson, Ariz. University of Arizona; 2008.

[54] Bazirete O, Nzayirambaho M, Umubyeyi A, Uwimana MC, Evans M. Influencing factors for prevention of postpartum hemorrhage and early detection of childbearing women at risk in Northern Province of Rwanda: beneficiary and health worker perspectives. BMC Pregnancy Childbirth. 2020;20(1):678. 33167935 10.1186/s12884-020-03389-7 PMC7654175

[55] Cubaka VK, Schriver M, Kayitare JB, Cotton P, Maindal HT, Nyirazinyoye L, et al. He should feel your pain’: Patient insights on patient-provider communication in Rwanda. Afr J Prim Health Care Fam Med. 2018;10(1):e1–11. 29781688 10.4102/phcfm.v10i1.1514 PMC5913761

[56] Påfs J, Musafili A, Binder-Finnema P, Klingberg-Allvin M, Rulisa S, Essén B. Beyond the numbers of maternal near-miss in Rwanda – a qualitative study on women’s perspectives on access and experiences of care in early and late stage of pregnancy. BMC Pregnancy Childbirth. 2016;16(1):257. 27590589 10.1186/s12884-016-1051-4 PMC5010768

[57] Engdawork K, Tadele G, Nahar P, Davey G, Zaman S. The effect of contextual factors on a health intervention against podoconiosis in Ethiopia. Front Trop Dis. 2024;5.

[58] Molla YB, Tomczyk S, Amberbir T, Tamiru A, Davey G. Podoconiosis in East and West Gojam Zones, Northern Ethiopia. PLoS Negl Trop Dis. 2012;6(7):e1744. 22816005 10.1371/journal.pntd.0001744 PMC3398962

[59] Kelemework A, Tora A, Amberbir T, Agedew G, Asmamaw A, Deribe K, et al. Why should I worry, since I have healthy feet?’ A qualitative study exploring barriers to use of footwear among rural community members in northern Ethiopia. BMJ Open. 2016;6(3):e010354. 27006343 10.1136/bmjopen-2015-010354 PMC4809094

[60] Ayode D, McBride CM, de Heer HD, Watanabe E, Gebreyesus T, Tora A, et al. A qualitative study exploring barriers related to use of footwear in rural highland ethiopia: implications for neglected tropical disease control. PLoS Negl Trop Dis. 2013;7(4):e2199. 23638211 10.1371/journal.pntd.0002199 PMC3636134

[61] Adler AJ, Randall T, Schwartz LN, Drown L, Matthews S, Pace LE, et al. What women want: A mixed-methods study of women’s health priorities, preferences, and experiences in care in three Rwandan rural districts. Int J Gynaecol Obstet. 2023;162(2):525–31. 36815725 10.1002/ijgo.14735

[62] Charles Mulindabigwi R, Josue M. The role of economic factors in the choice of medical providers in Rwanda. J Econ Behav Stud. 2016;8(2).

[63] Navale S, Habumugisha L, Amoroso C, Sayinzoga F, Gupta N, Hirschhorn LR. Exploring Drivers of Infant Deaths in Rural Rwanda Through Verbal Social Autopsy. Ann Glob Health. 2017;83(5–6):756–66. 29248092 10.1016/j.aogh.2017.10.029

[64] Ngang PN, Ntaganira J, Kalk A, Wolter S, Ecks S. Perceptions and beliefs about cough and tuberculosis and implications for TB control in rural Rwanda. Int J Tuberc Lung Dis. 2007;11(10):1108–13. 17945068

[65] Schurer JM, Dam A, Mutuyimana MT, Runanira DM, Nduwayezu R, Amuguni JH. At the hospital they do not treat venom from snakebites: A qualitative assessment of health seeking perspectives and experiences among snakebite victims in Rwanda. Toxicon X. 2022;14:100100. 35243331 10.1016/j.toxcx.2022.100100 PMC8885568

[66] Hategekimana F. Indigenous people, poverty and Community Based Health Insurance (CBHI) in Rwanda. Erasmus University Rotterdam; 2018.

[67] Schierenbeck I, Johansson P, Andersson LM, Krantz G, Ntaganira J. Collaboration or renunciation? The role of traditional medicine in mental health care in Rwanda and Eastern Cape Province, South Africa. Glob Public Health. 2018;13(2):159–72. 27712466 10.1080/17441692.2016.1239269

[68] Fothergill-Misbah N, Walker R, Kwasa J, Hooker J, Hampshire K. Old people problems, uncertainty and legitimacy: Challenges with diagnosing Parkinson’s disease in Kenya. Soc Sci Med. 2021;282:114148. 34153822 10.1016/j.socscimed.2021.114148

